# Saline Injections in Infantile Diarrhœa

**Published:** 1913-07-19

**Authors:** 


### SALINE INJECTIONS IN INFANTILE DIARRHŒA.

Some two years ago considerable excitement was aroused in the lay press by the publication of details concerning a method of treating infantile diarrhœa by subcutaneous injections of an expensive fluid consisting of diluted sea-water. A pretentious "clinic" was started by a small knot of medical practitioners to boom this treatment; and a good deal of publicity was achieved. Unsatisfactory as several aspects of this incident were, undoubtedly some good was achieved by re-directing the attention of the medical profession to the virtues of saline infusion in tiding small children through crises of serious depression. The fact had long been noted in text-books, but a good many practitioners had not absorbed it.

In Egypt acute infantile diarrhœa is an even greater scourge to the native population than it is in England; and the exceptionally abundant material thus provided has enabled Professor Day, of the Government School of Medicine, Cairo, to carry out an instructive series of comparative tests, the upshot of which he summarises in the *Practitioner*. Three saline fluids were used: Quinton's diluted sea-water, Ringer's solution, and Alexandrine sea-water diluted according to Quinton's method. All these solutions, as a matter of interest, are somewhat hypertonic. Professor Day concludes, on the strength of a most extensive experience of all the methods enumerated, that saline injections alone, without drugs, are capable of curing most cases of infantile diarrhœa; that Quinton's marine plasma has no definite superiority over artificial saline of the same strength; and that such hypertonic solutions are preferable to weaker ones. Nevertheless, even greater success can be obtained by medicinal treatment, which is preferable to saline injections as a routine treatment. Injections are valuable in proportion, as the loss of fluid by vomiting and diarrhœa exceeds the intake. They should be given before actual symptoms of collapse arise.

The hypothesis upon which the much-vaunted Quinton treatment is based has no rational basis whatever, and is palpably absurd. It is, therefore, not surprising to find that, even when saline injection is indicated, less costly fluids will answer the purpose just as well. We are not aware whether the tariff has been revised; but at the time of the boom this sea-water was being retailed at about the cost of champagne, a commercial proposition gratifying, no doubt, to the vendors, but less advantageous to everybody else.

